# Time to treatment with bridging intravenous alteplase before endovascular treatment:subanalysis of the randomized controlled SWIFT-DIRECT trial

**DOI:** 10.1136/jnis-2022-019207

**Published:** 2022-07-28

**Authors:** Thomas R Meinel, Johannes Kaesmacher, Lukas Buetikofer, Daniel Strbian, Omer Faruk Eker, Christophe Cognard, Pasquale Mordasini, Sandro Deppeler, Vitor Mendes Pereira, Jean François Albucher, Jean Darcourt, Romain Bourcier, Benoit Guillon, Chrysanthi Papagiannaki, Guillaume Costentin, Gerli Sibolt, Silja Räty, Benjamin Gory, Sébastien Richard, Jan Liman, Marielle Ernst, Marion Boulanger, Charlotte Barbier, Laura Mechtouff, Liqun Zhang, Gaultier Marnat, Igor Sibon, Omid Nikoubashman, Arno Reich, Arturo Consoli, David Weisenburger, Manuel Requena, Alvaro Garcia-Tornel, Suzana Saleme, Solène Moulin, Paolo Pagano, Guillaume Saliou, Emmanuel Carrera, Kevin Janot, Marti Boix, Raoul Pop, Lucie Della Schiava, Andreas Luft, Michel Piotin, Jean Christophe Gentric, Aleksandra Pikula, Waltraud Pfeilschifter, Marcel Arnold, Adnan Siddiqui, Michael T Froehler, Anthony J Furlan, René Chapot, Martin Wiesmann, Paolo Machi, Hans-Christoph Diener, Zsolt Kulcsar, Leo Bonati, Claudio Bassetti, Simon Escalard, David Liebeskind, Jeffrey L Saver, Urs Fischer, Jan Gralla

**Affiliations:** 1 Department of Neurology, Inselspital, Bern University Hospital, and University of Bern, Bern, Switzerland; 2 University Institute of Diagnostic and Interventional Neuroradiology, Inselspital, Bern University Hospital, and University of Bern, Bern, Switzerland; 3 CTU Bern, University of Bern, Bern, Switzerland; 4 Department of Neurology, Helsinki University Hospital and University of Helsinki, Helsinki, Finland; 5 Department of Neuroradiology, Hospices Civils de Lyon, Lyon, France; 6 Department of Diagnostic and Therapeutic Neuroradiology, Centre Hospitalier Universitaire de Toulouse, Toulouse, France; 7 Neuro Clinical Trial Unit, Department of Neurology, Inselspital, Bern University Hospital, and University of Bern, Bern, Switzerland; 8 Division of Neuroradiology and Division of Neurosurgery, Departments of Medical Imaging and Surgery, Toronto Western Hospital, University Health Network, University of Toronto, Toronto, Ontario, Canada; 9 Department of Neurology, Centre Hospitalier Universitaire de Toulouse, Toulouse, France; 10 Department of Diagnostic and Interventional Neuroradiology, Centre Hospitalier Universitaire de Nantes, Nantes Université, Nantes, France; 11 Department of Neurology, Centre Hospitalier Universitaire de Nantes, Nantes Université, Nantes, France; 12 Department of Radiology, CHU Rouen, Rouen, France; 13 Department of Neurology, CHU Rouen, Rouen, France; 14 Department of Diagnostic and Therapeutic Neuroradiology, CHRU-Nancy, Université de Lorraine, INSERM U1254, Nancy, France; 15 Department of Neurology, Stroke Unit, CHRU-Nancy, Université de Lorraine, INSERM U1116, Nancy, France; 16 Department of Neurology, Klinikum Nürnberg, Nürnberg, Germany; 17 Department of Diagnostic and Interventional Neuroradiology, University Medical Center Göttingen, Gottingen, Germany; 18 Deparment of Neurology, CHU Caen Normandie, University Caen Normandie, INSERM U1237, Caen, France; 19 Department of Neuroradiology, CHU Caen Normandie, University Caen Normandie, INSERM U1237, Caen, France; 20 Department of Vascular Neurology, Hospices Civils de Lyon, Lyon, France; 21 Department of Neurology, St George's University Hospital NHS Foundation Trust, London, UK; 22 Department of Interventional and Diagnostic Neuroradiology, CHU Bordeaux, University of Bordeaux, Bordeaux, France; 23 Stroke Unit, CHU Bordeaux, University of Bordeaux, Bordeaux, France; 24 Department of Neuroradiology, University Hospital RWTH Aachen, Aachen, Germany; 25 Department of Neurology, University Hospital RWTH Aachen, Aachen, Germany; 26 Department of Stroke and Diagnostic and Interventional Neuroradiology, Foch Hospital, Suresnes, France; 27 Stroke Unit, Department of Neurology, Hospital Vall d'Heborn, Barcelona, Spain; 28 Interventional Neuroradiology, Department of Radiology, Hospital Vall d'Heborn, Barcelona, Spain; 29 Department of Neuroradiology, CHU Limoges, Limoges, France; 30 Department of Neurology, CHU Reims, Reims, France; 31 Department of Neuroradiology, CHU Reims, Reims, France; 32 Service of Interventional and Diagnostic Radiology, Centre Hospitalier Universitaire Vaudois and University of Lausanne, Lausanne, Switzerland; 33 Department of Neurology, Hôpitaux Universitaires de Genève, Geneva, Switzerland; 34 Department of Diagnostic and Interventional Neuroradiology, Tours University Hospital, Tours, France; 35 Stroke Unit, Department of Neurosciences, University Hospital Germans Trias i Pujol, Barcelona, Spain; 36 Department of Interventional Neuroradiology, Strasbourg University Hospitals, Strasbourg, France; 37 Department of Neurology, Lille University Hospital, Lille, France; 38 Department of Neurology, University Hospital Zurich, Zurich, Switzerland; 39 Department of Neurology, Cereneo, Center for Neurology and Rehabilitation, Vitznau, Switzerland; 40 Department of interventional Neuroradiology, Fondation Rothschild Hospital, Paris, France; 41 Department of Neuroradiology, Brest University Hospital, Brest, France; 42 Department of Neurology, University Health Network - Toronto Western Hospital - University of Toronto, Toronto, Ontario, Canada; 43 Department of Neurology, University Hospital Frankfurt, Frankfurt, Germany; 44 Department of Neurosurgery, Jacobs School of Medicine and Biomedical Sciences, University at Buffalo, Buffalo, New York, USA; 45 Vanderbilt Cerebrovascular Program, Vanderbilt University Medical Center, Nashville, Tennessee, USA; 46 School of Medicine, Case Western Reserve University, Cleveland, Ohio, USA; 47 Department of Intracranial Endovascular Therapy, Alfried-Krupp Krankenhaus, Essen, Germany; 48 Department of Neuroradiology, Hôpitaux Universitaires de Genève, Geneva, Switzerland; 49 Department of Neuroepidemiology, Institute for Medical Informatics, Biometry and Epidemiology (IMIBE), Essen, Germany; 50 Department of Neuroradiology, University Hospital of Zurich, Zurich, Switzerland; 51 Department of Neurology, University Hospital Basel, Basel, Switzerland; 52 Department of Neurology and Comprehensive Stroke Center, David Geffen School of Medicine, UCLA, University of California, Los Angeles, California, USA

**Keywords:** Thrombolysis, Thrombectomy

## Abstract

**Background:**

We hypothesized that treatment delays might be an effect modifier regarding risks and benefits of intravenous thrombolysis (IVT) before mechanical thrombectomy (MT).

**Methods:**

We used the dataset of the SWIFT-DIRECT trial, which randomized 408 patients to IVT+MT or MT alone. Potential interactions between assignment to IVT+MT and expected time from onset-to-needle (OTN) as well as expected time from door-to-needle (DTN) were included in regression models. The primary outcome was functional independence (modified Rankin Scale (mRS) 0–2) at 3 months. Secondary outcomes included mRS shift, mortality, recanalization rates, and (symptomatic) intracranial hemorrhage at 24 hours.

**Results:**

We included 408 patients (IVT+MT 207, MT 201, median age 72 years (IQR 64–81), 209 (51.2%) female). The expected median OTN and DTN were 142 min and 54 min in the IVT+MT group and 129 min and 51 min in the MT alone group. Overall, there was no significant interaction between OTN and bridging IVT assignment regarding either the functional (adjusted OR (aOR) 0.76, 95% CI 0.45 to 1.30) and safety outcomes or the recanalization rates. Analysis of in-hospital delays showed no significant interaction between DTN and bridging IVT assignment regarding the dichotomized functional outcome (aOR 0.48, 95% CI 0.14 to 1.62), but the shift and mortality analyses suggested a greater benefit of IVT when in-hospital delays were short.

**Conclusions:**

We found no evidence that the effect of bridging IVT on functional independence is modified by overall or in-hospital treatment delays. Considering its low power, this subgroup analysis could have missed a clinically important effect, and exploratory analysis of secondary clinical outcomes indicated a potentially favorable effect of IVT with shorter in-hospital delays. Heterogeneity of the IVT effect size before MT should be further analyzed in individual patient meta-analysis of comparable trials.

**Trial registration number:**

URL: https://www.clinicaltrials.gov ; Unique identifier: NCT03192332

**WHAT IS ALREADY KNOWN ON THIS TOPIC**

- Overall, the randomized controlled trials on bridging thrombolysis before mechanical thrombectomy did not report any clear subgroup effects related to the time from symptom onset to randomization.

**WHAT THIS STUDY ADDS**

- This study found no clear evidence that patients with short onset-to-needle times benefited more from bridging thrombolysis. Exploratory analysis of secondary clinical outcomes indicated a potentially favorable effect of IVT associated with shorter in-hospital delays.

**HOW THIS STUDY MIGHT AFFECT RESEARCH, PRACTICE OR POLICY**

- This study sets methodological benchmarks for analyzing the heterogeneity of bridging thrombolysis effect size before mechanical thrombectomy in a meta-analysis of all randomized controlled trials on this topic. Neither onset-to-needle times nor door-to-needle times should influence treatment decisions regarding bridging thrombolysis until this meta-analysis is available.

## Introduction

Whether mechanical thrombectomy (MT) alone can be regarded as equally effective as MT combined with bridging intravenous thrombolysis (IVT+MT) for patients admitted directly to centers with endovascular treatment capability remains controversial.^1 2^ Two trials in Chinese patients demonstrated non-inferiority of MT alone,^3 4^ whereas three other trials failed to show non-inferiority.^5–7^ All these trials used generous non-inferiority margins, which are considerably less conservative than the proposed minimal clinically important difference or the margin considered to constitute reasonable comparability^8^ . The expedited recommendation of the European Stroke Organisation currently advises that patients admitted to MT-capable centers should undergo IVT+MT if eligible for both treatments.^9^

None of the individual subgroup analyses of these trials showed a significant difference regarding time from onset of symptoms to randomization (OTR). However, the point estimates indicated a potential time-dependent relationship between bridging IVT and functional outcome (table 1). In unselected stroke patients, the efficacy of IVT is known to be highly time-dependent.^10^ Therefore, we hypothesized that treatment delays might be an effect modifier regarding risks and benefits of IVT in patients enrolled in the SWIFT-DIRECT trial^7^ and that a more beneficial effect of IVT would be seen in patients with shorter treatment delays.

**Table 1:** Subgroup analysis of published randomized controlled trials

Study	Source	Outcome	Subgroup	acOR/aOR point estimate (95% CI)
MRCLEAN-NoIV^5^	online supplemental figure S3	Ordinal mRS score	OTR 13–77 min OTR 77–124 min OTR 124–734	0.75 (0.43 to 1.31) 0.67 (0.39 to 1.15) 1.00 (0.58 to 1.73)
DIRECT-MT^18^	online supplemental figure S4	Ordinal mRS score	OTR ≤125 min OTR 126–171 min OTR 172–210 min OTR >210 min	0.93 (0.54 to 1.61) 0.94 (0.54 to 1.64) 1.28 (0.74 to 2.22) 1.38 (0.79 to 2.40)
DEVT^19^	online supplemental eFigure 6	mRS 0–2	OTR <169 min OTR ≥169 min	0.97 (0.41 to 2.3) 2.25 (0.88 to 6.05)
SKIP^6^	Main paper figure 3	mRS 0–2	OTR <120 min OTR ≥120 min	0.77 (0.33 to 1.78) 1.33 (0.61 to 2.87)

In all trials a higher aOR/acOR favors withholding bridging IVT, while a lower aOR/acOR favors administering IVT before MT.

acOR, adjusted common OR; aOR, adjusted OR; IVT, intravenous thrombolysis; mRS, modified Rankin Scale; OTR, onset-to-randomization time.

10.1136/jnis-2022-019207.supp1 Supplementary data

This analysis aimed to assess a potential treatment effect heterogeneity of IVT+MT versus MT alone according to the overall delay (onset-to-needle, OTN) and in-hospital delays (door-to-needle, DTN) in terms of functional outcome, technical efficacy and safety outcomes. Additionally, if a heterogeneity of treatment effect was found, we intended to characterize the extent to which modification occurs and the time period during which adding IVT might confer significant benefits.

## Methods

### Reporting, data sharing, ethics

For this post-hoc sub-analysis of the randomized controlled SWIFT-DIRECT study (https://clinicaltrials.gov/ NCT03192332), we followed the CONSORT (Consolidated Standards of Reporting Trials) guidelines. The SWIFT-DIRECT dataset is not publicly available. However, de-identified data, together with a data dictionary, will be made accessible after ethics clearance and on submission of a reasonable request with a research plan to the corresponding author. Written informed consent was obtained from patients or their next of kin, with selected countries allowing delayed informed consent due to emergency circumstances. Approval was obtained from all relevant local ethics committees (central ethics Bern, ID 2017–00974).

### Study design and patients

SWIFT-DIRECT was an international, multicenter, randomized, open label, blinded endpoint (PROBE) trial assessing the non-inferiority of MT alone versus IVT+MT in patients presenting directly to one of 48 participating MT-capable stroke centers in Europe and Canada. The trial protocol^11^ and main results, including details of the methodology, have already been published.^7^ Patients were eligible if they had imaging-confirmed occlusion of the intracranial carotid artery and/or the first segment (M1) of the middle cerebral artery; were eligible to receive alteplase within 4.5 hours after they were last seen well; could undergo MT within 75 min of randomization; and had severe neurological deficits, defined as a National Institutes of Health Stroke Scale (NIHSS) score of ≥5. Patients with advanced dementia, significant pre-existing disabilities, and early severe tissue damage (Alberta Stroke Programme Early CT Score (ASPECTS) <5) were excluded. A total of 408 patients fulfilling those criteria were randomized (1:1 ratio) to undergo MT alone or IVT+MT (intravenous alteplase, 0.9 mg/kg of body weight). We included all patients in this post-hoc analysis.

### Time definitions

The goal of our study was to assess whether time to treatment was an effect modifier—that is, it would have an impact on the effect of IVT plus MT versus MT alone—with the idea that, depending on the time to treatment, additional IVT might show a benefit compared with MT alone. The time interval analyzed for the overall time delay was hence the expected OTN. This was defined as time from symptom onset or last known well to expected IVT bolus. It was calculated by adding the mean randomization-to-bolus-time to the onset-to-randomization value, for each patient in both the MT alone and the IVT+MT treatment groups.

For the in-hospital delay, the expected DTN was analyzed. This was defined as the time from arrival at the emergency department of the study hospital to the expected IVT bolus. It was calculated by adding to the door-to-randomization value, for each patient in both the MT alone and IVT+MT groups, the study mean for the randomization to bolus time. Those somewhat artificial time intervals were chosen since they represent the clinical scenario outside randomized controlled trials better than onset-to-randomization and door-to-randomization times. They are therefore easier to interpret and applicable to stroke centers. The study mean of DTN time was used due to the small sample sizes at individual centers and because there was little variation across sites. As a post-hoc sensitivity analysis, we used the individual time to IVT bolus administration for patients who received this treatment.

### Outcomes

Detailed definitions are available in the statistical analysis plan that was finalized and deposited^12^ before the analysis. The primary endpoint was functional independence, defined as modified Rankin Scale (mRS) ≤2 at 90 days. Secondary outcomes included mRS shift analysis, all-cause mortality, and time-to-reperfusion defined as expanded Thrombolysis In Cerebral Infarction (eTICI ≥2b). We also analyzed pharmacological efficacy (pre-interventional cross-sectional eTICI ≥2a (cs-eTICI), technical efficacy (eTICI ≥2b following device use) and safety outcomes (any and symptomatic intracranial hemorrhage, with the latter defined as ≥4 points worsening on the NIHSS within 24 hours).^13^

### Statistical analysis

An independent statistician (LB) organized, cleaned and analyzed the data according to the prespecified statistical analysis plan (see the online supplemental material). The intention-to-treat population was analyzed for a potential time- and IVT-arm-assignment interaction by comparing the outcomes in the IVT arm to the outcomes in the no IVT arm. Participant characteristics at randomization by time intervals from onset/last-seen-well to randomization were described using medians with IQR for continuous variables and proportions for discrete variables including all variables employed in any subsequent model.

The interaction was analyzed using logistic, linear or flexible parametric survival models for binary, continuous or time-to-event outcomes, respectively. For rare binary outcome, penalized maximum likelihood logistic regression (Firth method) was used. For the primary analysis, we analyzed the interaction term of the time interval (continuous variable)*IVT assignment. A linear relationship was used as default, but more flexible approaches (ie, fractional polynomials and linear splines) were also considered. For a secondary analysis, predefined time cut-offs were used with the rationale of the ‘golden hour’ for IVT (OTN 0–60 min vs 61–270 min),^14^ the Food and Drug Administration label for alteplase (0–180 min vs 181–270 min), and according to quartiles of OTN.^15^ Models were compared using Akaike and Bayesian information criteria. Interaction terms are reported with 95% confidence intervals (95% CI) and p values. Interpretation of p values of the interaction was based on the recommendations of the Instrument for assessing the Credibility of Effect Modification Analyses (ICEMAN) tool.^16^

Models were adjusted by the binary stratification variables and sex. Further covariate adjustments for baseline differences between early and late presenting patients were considered.

## Results

### Cohort characteristics

Between November 2017 and May 2021, 423 patients at 42 centers were randomized and 15 patients were excluded after randomization. Altogether, 201 patients were assigned to MT alone and 207 to IVT+MT. The allocated intervention was received by 402/408 patients with three crossovers in each treatment arm. Data completeness was almost perfect for mRS (one missing) and >95% for all other outcomes (see online supplemental figure S1 for the CONSORT flow-chart). The median age was 72 years (IQR 64–81), 209 (51.2%) were female, and the median NIHSS was 17 (13–20). The median OTN was 135 min (IQR 107–176) and the median DTN was 53 min (IQR 40–69). The expected median OTN and DTN were 142 (112–177) min and 54 (40–69) min in the IVT+MT group, and 129 (106–170) min and 51 (41–67) min in the MT alone group.

**Figure 1:**
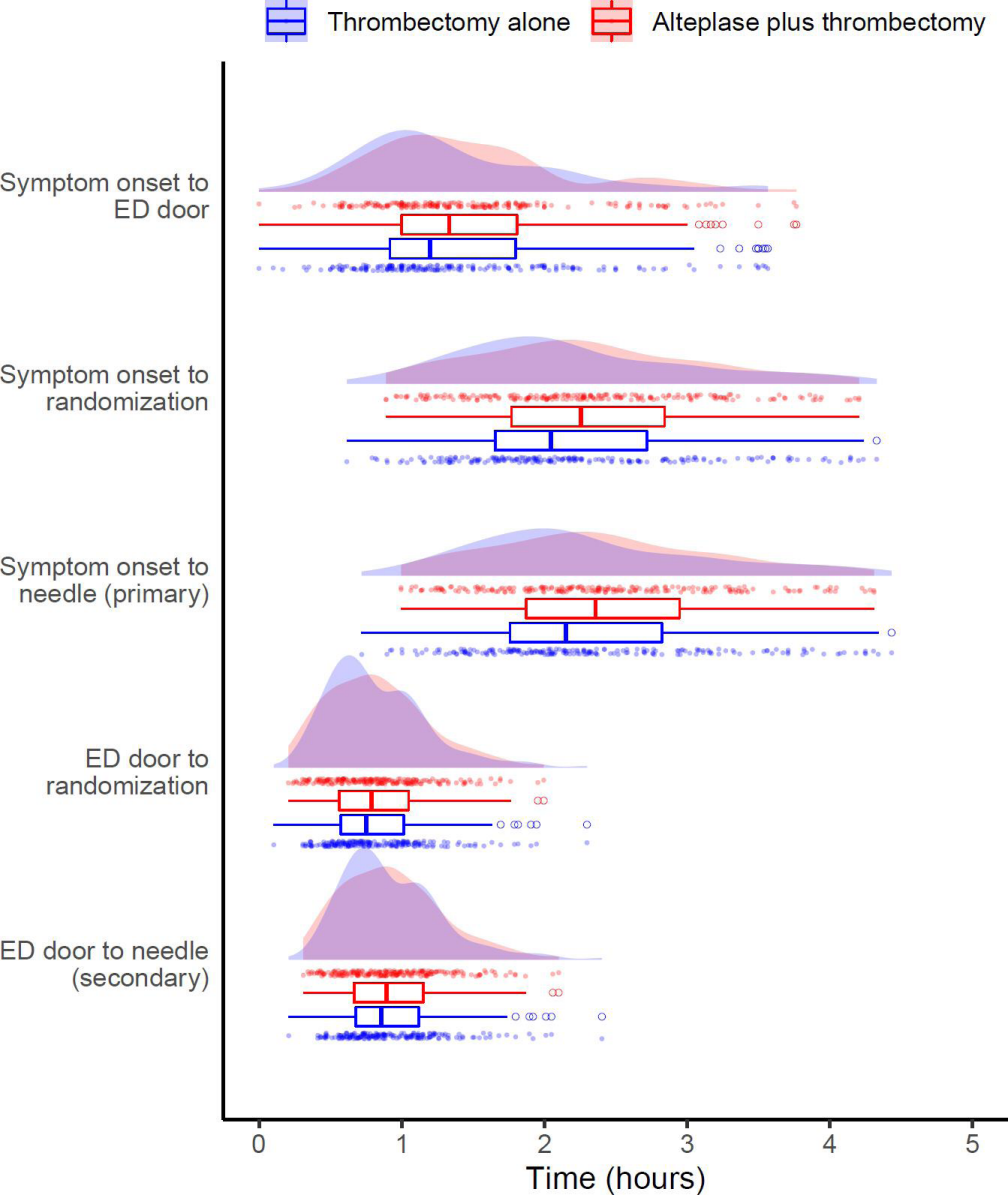
Distribution of time to treatment variables by randomization group. The median expected onset-to-needle time was 135 min (IQR 107–176) and the median expected door-to-needle time 53 min (IQR 40–69), without significant differences between both arms. The expected times were calculated as specified in the methods. For one patient the randomization date was interpolated. ED, emergency department.

Table 2 reports the baseline characteristics according to time delays of OTN; see online supplemental data table 1 for comparison according to DTN. Figure 1 depicts the distribution of time to treatment variables by randomization group.

**Table 2:** Selected baseline characteristics according to time from symptom onset to needle

	Time from symptom onset to needle				P value
	0–3 hours (n=316)		>3 hours (n=92)		
	N*		N*		
Age at inclusion (years), median (IQR)	316	72 (64–81)	92	74 (67–81)	0.27
Female sex, no. (%)	316	159 (50.3%)	92	50 (54.3%)	0.55
NIHSS, median (IQR)	316	17 (13–20)	92	17 (12–20)	0.8
Pre-stroke mRS, no. (%)	316		92		0.8
0		269 (85.1%)		77 (83.7%)	
1		46 (14.6%)		15 (16.3%)	
4		1 (0.3%)		0 (0.0%)	
Weight (kg), median (IQR)	293	75 (65–85)	89	75 (68–85)	0.81
Systolic blood pressure (mmHg), median (IQR)	312	147 (130–160)	91	149 (135–163)	0.58
Diastolic blood pressure (mmHg), median (IQR)	310	80 (70–90)	90	80 (71–90)	0.99
Heart rate (beats/min), median (IQR)	309	75 (64–88)	88	74 (63–86)	0.86
Previous ischemic stroke, no. (%)	304	30 (9.5%)	90	11 (12.0%)	0.55
Previous transient ischemic attack, no. (%)	300	14 (4.4%)	89	7 (7.6%)	0.28
History of hypertension, no. (%)	306	185 (58.5%)	92	54 (58.7%)	1
History of atrial fibrillation, no. (%)	299	28 (8.9%)	88	11 (12.0%)	0.42
History of hypercholesterolemia, no. (%)	298	98 (31.0%)	89	33 (35.9%)	0.38
Previous intracerebral hemorrhage, no. (%)	307	2 (0.6%)	90	0 (0.0%)	1
Prior myocardial infarction, no. (%)	301	37 (11.7%)	89	4 (4.3%)	0.047
Warfarin or other anticoagulant, no. (%)	316	11 (3.5%)	92	5 (5.4%)	0.37
Aspirin, no. (%)	316	84 (26.6%)	92	21 (22.8%)	0.5
Statin or other lipid lowering agent, no. (%)	316	91 (28.8%)	92	28 (30.4%)	0.79
Blood glucose level (mmol/L), median (IQR)	303	6.5 (5.7–7.5)	82	6.6 (5.9–7.6)	0.46
INR, median (IQR)	253	1.0 (1.0–1.1)	67	1.0 (1.0–1.1)	0.62
Platelet count (×10^9^/L), median (IQR)	314	225 (187–268)	91	228 (192–280)	0.28
Hemoglobin (g/L), median (IQR)	316	137 (125–146)	92	137 (123–147)	0.84
Glomerular filtration rate (mL/min), median (IQR)	316	76 (62–90)	92	74 (60–90)	0.89
Baseline imaging, no. (%)	316		92		<0.001
CT		177 (56.0%)		28 (30.4%)	
MRI		137 (43.4%)		63 (68.5%)	
Both		2 (0.6%)		1 (1.1%)	
ASPECTS (core lab), median (IQR)	315	8.0 (7.0–9.0)	92	8.0 (6.0–8.5)	0.004
Baseline intracranial occlusion site, no. (%)	316		92		0.99
Distal ICA - I		12 (3.8%)		4 (4.3%)	
Distal ICA - I and M1		2 (0.6%)		0 (0.0%)	
Distal ICA - L		41 (13.0%)		13 (14.1%)	
Distal ICA - T		37 (11.7%)		8 (8.7%)	
Distal M1		96 (30.4%)		29 (31.5%)	
Distal M2		3 (0.9%)		1 (1.1%)	
Proximal M1		110 (34.8%)		34 (37.0%)	
Proximal M2		15 (4.7%)		3 (3.3%)	
Tandem lesion, no. (%)	316	45 (14.2%)	92	18 (19.6%)	0.25

ASPECTS, Alberta Stroke Programme Early CT Score; ICA, internal carotid artery; INR, International normalized ratio; mRS, modified Rankin Scale; N*, number of patients with non-missing data; NIHSS, National Institutes of Health Stroke Scale.

### Delay from onset (OTN)

**Figure 2:**
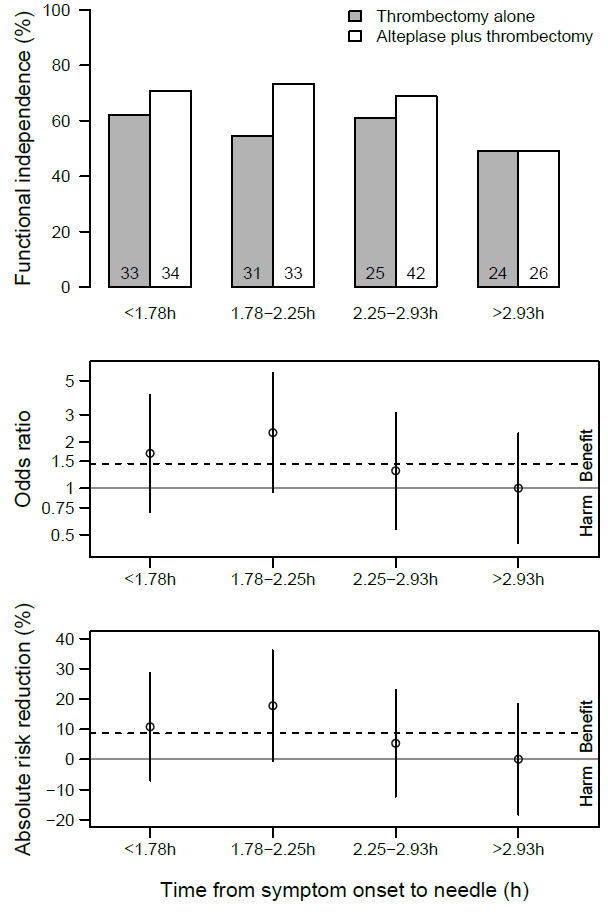
Benefit according to quartiles of expected time from symptom onset to last known well to IVT bolus. (Top panel) Event rates of functional independence (%, (N)). (Middle panel) Odds ratios of IVT+MT versus MT alone with the dashed line indicating the marginal effect over all categories and the gray line the zero effect. (Bottom panel) Absolute risk reduction for IVT+MT versus MT alone. Benefit and harm refer to combination of IVT+MT versus MT alone. IVT, intravenous thrombolysis; MT, mechanical thrombectomy.

We found no evidence that the effect of bridging IVT on functional independence was modified by the delay of OTN. The odds for functional independence in patients treated with alteplase plus thrombectomy versus thrombectomy alone numerically decreased by 0.76 (95% CI 0.45 to 1.30, p=0.32) per hour of OTN delay. Similar results were obtained when assuming a dichotomous effect (adjusted odds ratio (aOR) of >3 hours vs 0–3 hours 0.64, 95% CI 0.24 to 1.72, p=0.37), across quartiles (see figure 2) or when using linear splines. Models fitted best when OTN was included as a linear effect and consistent with the sensitivity analysis using the individual times to IVT bolus administration (see online supplemental table S2).

There was no significant interaction of OTN and bridging IVT assignment in terms of the safety outcomes or the pharmacological and technical efficacy (see table 3).

**Table 3:** Interaction analysis regarding primary and secondary outcomes according to overall and in-hospital delays

Time	Outcome category	Outcome	aOR for MT alone per 1 hour delay with 95% CI	aOR of interaction per 1 hour delay with 95% CI*
Onset-to-needle* time: Expected time from symptom onset or last known well to IVT bolus	**Efficacy**	mRS 0–2 (primary), day 90	0.86, 0.60 to 1.23	0.76, 0.45 to 1.30
		mRS decrease (better outcome), day 90	0.82, 0.60 to 1.12	0.90, 0.58 to 1.39
		Mortality, day 90	1.57, 0.91 to 2.70	0.98, 0.42 to 2.32
	**Safety**	Any ICH on 24 hours imaging	1.35, 0.93 to 1.97	1.33, 0.78 to 2.27
		Symptomatic ICH on 24 hours imaging	1.15, 0.42 to 3.17	0.66, 0.17 to 2.65
	**Pharmacological efficacy**	Pre-interventional reperfusion success (cs-eTICI ≥2a)	0.99, 0.40 to 2.42	1.56, 0.54 to 4.49
		Time-to-reperfusion	0.73, 0.60 to 0.89	1.24, 0.94 to 1.62,
		Final reperfusion success (cs-eTICI ≥2b)	0.78, 0.44 to 1.37	1.04, 0.36 to 3.02
Door-to-needle* time: Expected time from arrival at the emergency department door to IVT bolus	**Efficacy**	mRS 0–2 (primary), day 90	1.47, 0.60 to 3.56	0.48, 0.14 to 1.62
		mRS decrease (better outcome), day 90	1.88, 0.91 to 3.88	0.36, 0.13 to 0.99
		Mortality, day 90	0.11, 0.02 to 0.66	17.8, 1.8 to 174.9
	**Safety**	Any ICH on 24 hours imaging	0.99, 0.40 to 2.43	0.95, 0.28 to 3.24
		Symptomatic ICH on 24 hours imaging	0.73, 0.05 to 10.74	4.60, 0.19 to 114.10
	**Pharmacological efficacy**	Pre-interventional reperfusion success (cs-eTICI ≥2a)	2.26, 0.36 to 14.38	0.63, 0.07 to 6.06
		Time-to-reperfusion	0.40, 0.26 to 0.63	0.88, 0.47 to 1.64
		Final reperfusion success (cs-eTICI ≥2b)	1.69, 0.37 to 7.81	0.56, 0.05 to 6.83

*The aOR indicates the interaction term of assignment to IVT+MT (as compared with MT alone) and 1 hour delay and group assignment assuming a linear effect. The OR for MT alone gives the change in the odds for functional independence per additional hour delay. The interaction refers to change in the treatment effect (odds for functional independence of IVT plus MT vs MT alone) per additional hour delay.

aOR, adjusted OR; cs-eTICI, cross-sectional expanded Thrombolysis In Cerebral Infarction; ICH, intracranial hemorrhage; IVT, intravenous thrombolysis; mRS, modified Rankin Scale; MT, mechanical thrombectomy.

### In-hospital delay (DTN)

We also found no evidence that the effect of bridging IVT on functional independence is modified by the in-hospital delay. No heterogeneity was observed, including when assuming a dichotomous effect of DTN (aOR of >1 hour vs 0–1 hour 1.37, 95% CI 0.74 to 2.53). Similarly, across quartiles, there was no interaction of DTN and IVT assignment in terms of the primary outcome.

The adjusted odds for a favorable mRS shift numerically increased by 1.88 (95% CI 0.91 to 3.88) per 1 hour decrease of DTN resulting in a significant interaction with IVT assignment (aOR 0.36, 95% CI 0.13 to 0.99, p=0.047). In parallel, the mortality analysis (aOR 17.8, 95% CI 1.8 to 174.9, p for interaction 0.011) provided some evidence for a more beneficial effect of IVT when in-hospital delays were short (table 3).

## Discussion

This post-hoc analysis of the SWIFT-DIRECT trial found no clear evidence that patients with short OTN benefited more from bridging IVT. Exploratory analysis of secondary clinical outcomes indicated a potentially favorable effect of IVT associated with shorter in-hospital delays.

For patients qualifying for IVT without MT, earlier treatment is associated with increased proportional benefits, with potential harms only evident beyond the established 4.5 hour limit.^17^ For patients who received bridging IVT before MT, the randomized controlled trials on this topic have reported no clear subgroup effects related to the time from symptom onset to randomization. Also, our nuanced sub-analysis of the randomized SWIFT-DIRECT trial detected no heterogeneity of treatment effect. Our model fit was best when OTN was handled as a continuous variable (ie, assumption of a linear effect). The point estimate (aOR 0.76, 95% CI 0.45 to 1.30) crossed the zero effect line indicating potential harm at around 4 hours after symptom onset for the dichotomized functional independence and beyond 4 hours for the mRS shift analysis (aOR 0.90, 95% CI 0.58 to 1.39). Nevertheless, given the point estimates of all trials on this topic,^5 6 18 19^ the pathophysiology of ischemic stroke and IVT, as well as the low power of the subgroup analysis,^15^ it is possible that we missed a clinically important effect. Hence, this analysis should be repeated in an individual patient meta-analysis of comparable trials on bridging IVT.

No interaction could be detected with the secondary safety outcomes, and pharmacological and technical efficacy. However, a sub-analysis of the DEVT trial recently reported an association of bridging IVT with increased early reperfusion when MT was delayed more than approximately half an hour.^20^

Analysis of in-hospital delays revealed a potential heterogeneity of treatment effect of IVT regarding mortality and mRS shift analysis, with a larger proportional benefit seen when DTN was shorter. However, the credibility of those subgroup effects is unclear because of multiple testing and hence, this finding might be due to chance.^16^ Nevertheless, since the anticipated direction of the effect and the pathophysiology support such heterogeneity, we suggest a re-analysis in an individual patient meta-analysis of the trials mentioned above. In a bigger dataset, potentially relevant subgroups such as tandem lesions should be specifically addressed.^2^

The meta-analysis of the trials on MT^21^ also found a time-to-treatment interaction for in-hospital delays, but not for overall delays from symptom onset. Possible reasons include a stronger association of in-hospital delays with outcome, the time-reset effect of imaging-based inclusion,^22^ uncertain trustworthiness of pre- versus in-hospital time workflow information, and non-linear ischemic core growth over time.^23 24^

### Strengths and limitations

Strengths include good overall data quality within the setting of the randomized prospective international multicenter SWIFT-DIRECT trial and a prespecified, deposited statistical analysis plan with defensive interpretation according to recommendations for subgroup analysis of randomized trials. Limitations are mainly related to the fact that the study was neither designed nor powered to detect an interaction effect—that is, assuming the observed correlations from the main study, odds ratios lower than 0.6 would be necessary to reach a power of 80%. Since imaging selection (ASPECTS) was used in the enrolled patients, the time effects observed are likely to be less pronounced than those that would occur in the overall population of patients with large-vessel occlusion in the absence of imaging selection.

## Conclusions

This subgroup analysis found no evidence that the effect of bridging IVT on functional independence is modified by overall or in-hospital treatment delays. Considering the low statistical power of this subgroup analysis, a clinically important effect could have been missed. Nevertheless, exploratory analysis regarding secondary clinical outcomes indicated a potentially favorable effect of IVT associated with shorter in-hospital delays. Until further evidence regarding potential heterogeneity of the IVT effect size before MT becomes available from individual patient meta-analysis of comparable trials, IVT should be given to eligible patients and neither OTN nor DTN should influence treatment decisions regarding bridging IVT.

10.1136/jnis-2022-019207.supp2 Supplementary data

## Data Availability

Data are available upon reasonable request. De-identified data, together with a data dictionary, will be made accessible after ethics clearance and upon submission of a reasonable request with a research plan to the corresponding author.
