# Supplementary material for: Time to treatment with bridging intravenous alteplase before endovascular treatment:subanalysis of the randomized controlled SWIFT-DIRECT trial
Source: J Neurointerv Surg. 2022 Jul 28;15(e1):e102–10. doi: 10.1136/jnis-2022-019207 (PMC10715486; doi:10.1136/jnis-2022-019207)
Supplement: Supplementary data [file jnis-2022-019207supp001.pdf]

Online Supplement

Time to Treatment With Bridging Intravenous Alteplase Prior to Endovascular Treatment – subanalysis of the randomized controlled SWIFT-DIRECT trial

Table S1 - Baseline characteristics by dichotomized time from ED door to needle (DTN).

	Time from ED door to needle				
		0-1 h (N = 255)		*>1 h (N = 153)*	*P-value*
	N*		N*		
Age at inclusion - median (IQR)	255	72 (64, 81)	153	74 (64, 81)	0.36
Female sex - no. (%)	255	123 (48.2%)	153	86 (56.2%)	0.13
NIHSS - median (IQR)	255	17 (14, 20)	153	17 (12, 20)	0.11
Pre-stroke mRS - no. (%)	255		153		0.45
0		212 (83.1%)		134 (87.6%)	
1		42 (16.5%)		19 (12.4%)	
4		1 (0.4%)		0 (0.0%)	
Weight (kg) - median (IQR)	234	75 (65, 85)	148	75 (66, 85)	0.76
Systolic blood pressure (mmHg) - median (IQR)	251	149 (134, 163)	152	145 (128, 160)	0.10
Diastolic blood pressure (mmHg) - median (IQR)	248	80 (70, 90)	152	80 (71, 90)	0.92
Heart rate (beats per minute) - median (IQR)	246	75 (64, 86)	151	73 (63, 90)	0.84
Previous ischemic stroke - no. (%)	243	26 (10.2%)	151	15 (9.8%)	1.00
Previous transient ischemic attack - no. (%)	239	13 (5.1%)	150	8 (5.2%)	1.00
History of hypertension - no. (%)	249	152 (59.6%)	149	87 (56.9%)	0.60
History of atrial fibrillation - no. (%)	238	20 (7.8%)	149	19 (12.4%)	0.16
History of hypercholesterolemia - no. (%)	238	82 (32.2%)	149	49 (32.0%)	1.00
Previous intracerebral hemorrhage - no. (%)	247	2 (0.8%)	150	0 (0.0%)	0.53
Prior myocardial infarction - no. (%)	241	25 (9.8%)	149	16 (10.5%)	0.87
Warfarin or other anticoagulant - no. (%)	255	8 (3.1%)	153	8 (5.2%)	0.30
Aspirin - no. (%)	255	71 (27.8%)	153	34 (22.2%)	0.24
Statine or other lipid lowering agent - no. (%)	255	74 (29.0%)	153	45 (29.4%)	1.00
Blood glucose level (mmol/L) - median (IQR)	245	6.5 (5.8, 7.5)	140	6.7 (5.8, 7.5)	0.57
International normalized ratio (INR) - median (IQR)	216	1.0 (1.0, 1.1)	104	1.0 (1.0, 1.1)	0.045
Platelet count x 10 E9(G/L) - median (IQR)	254	225 (187, 268)	151	231 (189, 280)	0.25
Hemoglobin (g/L) - median (IQR)	255	136 (125, 146)	153	138 (125, 147)	0.65
Glomerular filtration rate (mL/min) - median (IQR)	255	76 (63, 90)	153	77 (58, 90)	0.75
Baseline imaging - no. (%)	255		153		<0.001
CT		164 (64.3%)		41 (26.8%)	
MRI		90 (35.3%)		110 (71.9%)	
both		1 (0.4%)		2 (1.3%)	
ASPECTS (core lab) - median (IQR)	254	8.0 (7.0, 9.0)	153	8.0 (7.0, 9.0)	0.20
Baseline intracranial occlusion site - no. (%)	255		153		0.53
Distal ICA - I		10 (3.9%)		6 (3.9%)	
Distal ICA - I and M1		1 (0.4%)		1 (0.7%)	
Distal ICA - L		29 (11.4%)		25 (16.3%)	
Distal ICA - T		31 (12.2%)		14 (9.2%)	
Distal M1		72 (28.2%)		53 (34.6%)	
Distal M2		3 (1.2%)		1 (0.7%)	
Proximal M1		97 (38.0%)		47 (30.7%)	
Proximal M2		12 (4.7%)		6 (3.9%)	
Tandem lesion - no. (%)	255	44 (17.3%)	153	19 (12.4%)	0.21

N: number of patients with non-missing data*

Table S2 - Sensitivity analysis using individual time to IVT bolus for patients who received IVT.

Time	Outcome category	Outcome	aOR for MT alone per 1-hour delay with 95%-CI*	aOR of interaction per 1-hour delay with 95%-CI**
**Onset-to-Needle time:** Time from symptom onset or last known well to IVT bolus for patients receiving IVT and expected time for those not receiving IVT	Efficacy	mRS 0-2 (primary), day 90	0.85, 0.60 - 1.22	0.72, 0.43 - 1.22
		mRS decrease (better outcome), day 90	0.80, 0.59 - 1.10	0.86, 0.56 - 1.33
		Mortality, day 90	1.61, 0.95 - 2.73	1.01, 0.43 - 2.37
	Safety	Any ICH on 24h imaging	1.31, 0.91 - 1.90	1.42, 0.83 - 2.42
		Symptomatic ICH on 24h imaging	1.15, 0.43 - 3.11	0.67, 0.17 - 2.61
	Pharmacological efficacy	Preinterventional reperfusion success (cs-eTICI≥2a)	0.99, 0.41 - 2.43	1.66, 0.57 - 4.77
		Time-to-reperfusion	0.73, 0.60 - 0.89	1.14, 0.87 - 1.51
		Final reperfusion success (cs-eTICI≥2b)	0.78, 0.45 - 1.37	0.94, 0.33 - 2.73
**Door-to-Needle time:** Time from arrival at the emergency department door to IVT bolus for patients receiving IVT and expected time for those not receiving IVT	Efficacy	mRS 0-2 (primary), day 90	1.39, 0.57 - 3.39	0.43, 0.14 - 1.37
		mRS decrease (better outcome), day 90	1.70, 0.82 - 3.52	0.35, 0.14 - 0.91
		Mortality, day 90	0.19, 0.03 - 1.01	12.4, 1.4 - 110.5

	Safety	Any ICH on 24h imaging	0.87, 0.35 - 2.17	1.18, 0.36 - 3.80
		Symptomatic ICH on 24h imaging	0.71, 0.05 - 11.03	3.75, 0.16 - 85.94
	Pharmacological efficacy	Preinterventional reperfusion success (cs-eTICI≥2a)	2.30, 0.36 - 14.55	0.71, 0.08 - 5.98
		Time-to-reperfusion	0.39, 0.25 - 0.61	0.88, 0.48 - 1.60
		Final reperfusion success (cs-eTICI≥2b)	1.75, 0.37 - 8.22	0.39, 0.04 - 3.91

mRS: modified Rankin Scale; aOR: adjusted odds ratio; ICH: intracranial hemorrhage

*The aOR for MT alone indicates the effect of one hour delay on the outcome in the MT group assuming a linear effect.

**The aOR indicates the interaction term of assignment to IVT+MT (as compared to MT alone) and one hour delay and group assignment assuming a linear effect.

Figure S1 – CONSORT study flow-chart

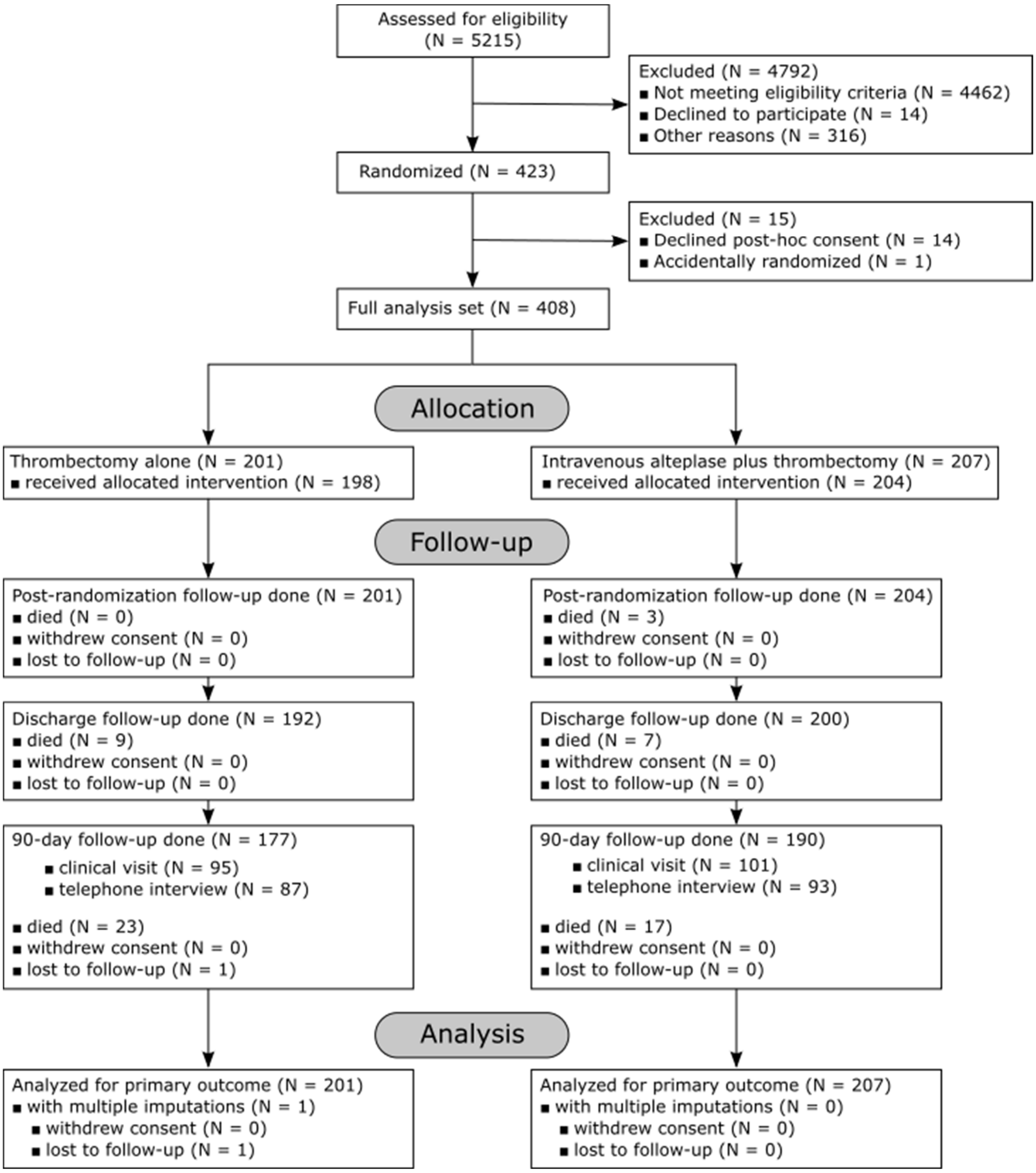

Other reasons for exclusion were absence of the study team (n=237), inclusion in a competing trial (n=10), deemed not suitable for Solitaire stent-retriever/thrombectomy by the local operator (n=23), out of working hours presentation (n=41), and an individual decision by the stroke consultant to prioritize thrombolysis (n=5).
